# Tuberculosis-associated mortality and risk factors for HIV-infected population in Ethiopia: a systematic review and meta-analysis

**DOI:** 10.3389/fpubh.2024.1386113

**Published:** 2024-07-22

**Authors:** Fassikaw Kebede Bizuneh, Tsehay Kebede Bizuneh, Seteamlak Adane Masresha, Atitegeb Abera Kidie, Mulugeta Wodaje Arage, Nurye Sirage, Biruk Beletew Abate

**Affiliations:** ^1^College of Health Sciences, Woldia University, Woldia, Ethiopia; ^2^Faculties of Social Science, Geography department, Bahir Dare University, Bahir Dare, Ethiopia

**Keywords:** mortality, tuberculosis, HIV, antiretroviral, children, adult, Ethiopia, population

## Abstract

**Background:**

Despite the effectiveness of antiretroviral therapy in reducing mortality from opportunistic infections among people living with HIV (PLHIV), tuberculosis (TB) continues to be a significant cause of death, accounting for over one-third of all deaths in this population. In Ethiopia, there is a lack of comprehensive and aggregated data on the national level for TB-associated mortality during co-infection with HIV. Therefore, this systematic review and meta-analysis aimed to estimate TB-associated mortality and identify risk factors for PLHIV in Ethiopia.

**Methods:**

We conducted an extensive systematic review of the literature using the Preferred Reporting of Systematic Review and Meta-Analysis (PRISMA) guidelines. More than seven international electronic databases were used to extract 1,196 published articles from Scopus, PubMed, MEDLINE, Web of Science, HINARY, Google Scholar, African Journal Online, and manual searching. The pooled mortality proportion of active TB was estimated using a weighted inverse variance random-effects meta-regression using STATA version-17. The heterogeneity of the articles was evaluated using Cochran’s Q test and *I*^2^ statistic test. Subgroup analysis, sensitivity analysis, and Egger’s regression were conducted to investigate publication bias. This systematic review is registered in Prospero with specific No. CRD42024509131.

**Results:**

Overall, 22 individual studies were included in the final meta-analysis reports. During the review, a total of 9,856 cases of TB and HIV co-infection were screened and 1,296 deaths were reported. In the final meta-analysis, the pooled TB-associated mortality for PLHIV in Ethiopia was found to be 16.2% (95% CI: 13.0–19.2, *I*^2^ = 92.9%, *p* = 0.001). The subgroup analysis revealed that the Amhara region had a higher proportion of TB-associated mortality, which was reported to be 21.1% (95% CI: 18.1–28.0, *I*^2^ = 84.4%, *p* = 0.001), compared to studies conducted in Harari and Addis Ababa regions, which had the proportions of 10% (95% CI: 6–13.1%, *I*^2^ = 83.38%, *p* = 0.001) and 8% (95% CI: 1.1–15, *I*^2^ = 87.6%, *p* = 0.001), respectively. During the random-effects meta-regression, factors associated with co-infection of mortality in TB and HIV were identified, including WHO clinical stages III & IV (OR = 3.01, 95% CI: 1.9–4.7), missed co-trimoxazole preventive therapy (CPT) (OR = 1.89, 95% CI: 1.05–3.4), and missed isoniazid preventive therapy (IPT) (OR = 1.8, 95% CI: 1.46–2.3).

**Conclusion:**

In Ethiopia, the mortality rate among individuals co-infected with TB/HIV is notably high, with nearly one-fifth (16%) of individuals succumbing during co-infection; this rate is considered to be higher compared to other African countries. Risk factors for death during co-infection were identified; the included studies examined advanced WHO clinical stages IV and III, hemoglobin levels (≤10 mg/dL), missed isoniazid preventive therapy (IPT), and missed cotrimoxazole preventive therapy (CPT) as predictors. To reduce premature deaths, healthcare providers must prioritize active TB screening, ensure timely diagnosis, and provide nutritional counseling in each consecutive visit.

**Systematic review registration:**

Trial registration number in Prospero =CRD42024509131 https://www.crd.york.ac.uk/prospero/display_record.php?RecordID=509131.

## Introduction

People living with the human Immune deficiency virus (PLHIV) are more susceptible to tuberculosis (TB), which is a leading cause of mortality (1, 2). There is a strong synergy between HIV infection and TB, as PLHIV are at high risk of dying from TB, and HIV infection is the biggest risk factor for active TB incidence due to declining cellular immunity and increased endogenous reactivation of latent TB bacilli in the lungs (3, 4).

Globally in 2020, 1.2 million TB deaths from HIV-negative people and 208,000 deaths from HIV-positive individuals were reported (5). TB infection remains the leading cause of morbidity and mortality for PLHIV worldwide (6). In Sub-Saharan Africa (SSA) countries, the incidence of new TB-associated cases and new mortality cases were reported to be 2,017 cases/100,000 patients per year (7) and 25 cases/100 patients per year, respectively (8–10). Global TB reports of 2022 indicated that the African continent accounted for 1.5 million TB deaths, with 14.30% of those being co-infected deaths among PLHIV (1).

Previous studies have identified several factors contributing to mortality during TB/HIV co-infection, including missed isoniazid preventive therapy (IPT), poor adherence to antiretroviral therapy (ART), acute malnutrition, late diagnosis, the influence of HIV on clinical presentation and diagnosis, immune reconstitution inflammatory syndrome (IRIS), interruptions in drug treatment, and loss to follow-up (LTFU), presence of multiple comorbidities, hepatotoxicity, low body mass index (BMI), and poor medication adherence, were prominent predictors for premature death among PLHIV (11–13). However, having a CD4 count of ≤200 cells/mL serves as a proxy indicator for premature death during twine infection (during TB and HIV co-infection) (6, 14). The finding of a previous retrospective cohort study involving 4,210 co-infected patients in Ethiopia revealed that more than 72.1% of death cases occurred when the CD4 count was ≤200 cells/mL (15).

Reducing mortality during co-infection can be achieved by implementing effective public health policies, such as comprehensive case screening and timely treatment, along with nutritional counseling (3, 16), and concurrent administration of IPT with highly active antiretroviral therapy (HAART) could decrease active TB occurrences by ≥90% (17). However, perceived stigma during health care provision was attributed to 63.7% incidence of common mental health disorders, indirectly contributing to loss to follow-up and premature death (16, 18, 19). In Ethiopia, deaths from the co-infection varied both at the population level and across each region, with 23.01 cases per 100 person-years in Tigray for adults (20), 17.15 cases per 100 person-years for children in Southern Ethiopia (21), and 10.9 cases per 100 person-years in Amhara region (17).

In Ethiopia, despite improvements in ART coverage, implementation of IPT, and collaborative efforts in TB/HIV management, the rates of co-infection and associated mortality remain high. Limited systematic review and meta-analysis studies exist on the mortality for TB/HIV-co-infected population in Ethiopia, and there is a lack of aggregated data on TB-associated mortality during co-infection; this meta-analysis aimed to estimate the pooled TB-associated mortality and identify risk factors for people living with HIV in Ethiopia. This systematic review and meta-analysis report provide evidence on TB mortality rates in both adults and children, regional estimates of mortality rates, and associated risk factors for vulnerable populations.

## Methods

### Study setting and periods

This systematic review and meta-analysis study was conducted from 1 January 2013 to 30 December 2022 over a 10-year-period in Ethiopia. The study covered nine regions in Ethiopia, namely, Tigray, Afar, Amhara, Oromia, SNNR, Somalia, Gambella, Benishangul Gumuz, and Harari, and two city administrative areas (11).

### Protocol registration and searching strategy

Based on the information provided, the protocol of the study can be accessed through the following web address: https://www.crd.york.ac.uk/prospero/#recordDetails. The protocol registration number for the study is CRD42024509131. The Preferred Reporting Items for Systematic Reviews and Meta-Analysis (PRISMA-2020) guideline has been utilized to report the findings of this study (Supplementary Table S1) (22).

### Eligibility criteria

#### Inclusion criteria

This systematic review and meta-analysis report included articles with defined outcomes of any type of TB-associated death among PLHIV based on the following inclusion criteria: (1) scientific papers reporting co-infections of TB and HIV leading to death among PLHIV in Ethiopia; (2) articles containing defined estimates of death, including either incidence proportion or prevalence proportion of TB and HIV co-infection in their final reports; (3) observational studies (including cross-sectional, cohort, and case control studies) published in English, without time restrictions; and (4) study subjects including either young adults or adolescents or adult population living with HIV in Ethiopia. Only studies that met the inclusion criteria were eligible for this meta-analysis.

#### Exclusion criteria

Studies that reported lacking abstracts and/or full-text, anonymous reports, editorials, and qualitative studies were excluded from the analysis. Furthermore, before the analysis, unfitted articles without a journal name and/or author, articles that lacked the year of publication, and those with citations but without abstracts and/or full text available were excluded from the final meta-analysis.

### Outcome ascertainment

This systematic review and meta-analysis had two main objectives. The first objective was the estimation of the proportion of death during TB and HIV co-infection (TB can be PTB or EXPTB) among HIV-infected population after HIV care was started in Ethiopia. The second objective was identifying risk factors for death during co-infection treatment, which was achieved by collecting significant predictors reported in the included studies with their adjusted odds ratio and their respective 95% CIs extracted and then calculated during the meta-analysis.

### Information sources for articles searching

Articles were retrieved from seven databases, namely PubMed (*N* = 784), Scopus (*N* = 68), MEDLINE (*N* = 43), Web of Science (*N* = 39), HINARY (*N* = 161), Google Scholar (*N* = 70), and African Journal Online (*N* = 22), without time restriction for inclusion. However, the search was limited by studies conducted in Ethiopia and articles published only in the English language.

### Data searching

The search strategy employed included specified eligible criteria and the following MeSH terms: (1) mortality, (2) incidence, (3) tuberculosis, (4) HIV infection, (5) individuals, (6) children, (7) adults, and (8) Ethiopia; the included articles were extracted. The authors employed the following search terms to retrieve relevant studies from databases: “Epidemiology” OR “prevalence” OR “Incidence” AND “Death,” OR “Mortality,” OR” Case fatality” AND “Tuberculosis,” OR “Pulmonary TB,” OR “Disseminated TB,” OR “TB lymphadenitis,” AND “HIV,” OR “AIDS,” AND “Children” OR “Pediatrics” OR “Infant” OR “Adult” OR “Population” AND “Ethiopia.” The search process involved three authors (FK, DT, and TK) who selected the most pertinent studies based on predefined criteria.

### Quality assessment and appraisal procedures

Four authors (FK, BB, SA, and AA) independently extracted the data and evaluated the quality of each study by determining the eligibility of the titles and abstracts of the studies after removing duplicates. Any disagreement or uncertainty that arose during the article extraction process was resolved by discussion. These reviewers assessed the full-text of the studies; if one or more of them believed a study could be significant, it qualified for inclusion after the study was carefully examined for its titles, abstracts, and full text. Three authors (MW, NS, and BB) used a Microsoft Excel spreadsheet to extract the specifics of each study. After through discussion with a third-party reviewer (FK and AA), the disagreement during data extraction was resolved by consensus. All eligible studies were approved with the agreement of all authors, and any differences were worked out through discussion until a consensus was reached. Following the agreement, information about principal investigators, year of publication, study period, study setting, study population, and sample size was retrieved from the identified articles. The authors of the study conducted a comprehensive assessment of the risk of bias in all included studies. This assessment was performed by SA, TK, and BB and involved evaluating and screening the studies. The Joanna Briggs Institute of Critical Appraisal (JBI) checklist was utilized to evaluate the quality of the studies, and the findings from this assessment were incorporated into the final meta-analysis. To gather the relevant published article citations, the authors employed Endnote version 8 and exported and collected all potentially suitable citations, ensuring that any duplication was removed during the selection and screening processes. A team of five independent reviewers (FK, BB, NS, MW, and TK) first reviewed the abstracts of the publications to determine their suitability. Subsequently, they evaluated the full-text articles. The quality of the included articles was assessed using the JBI checklist for cohort-study design evaluation criteria, which consists of 11 critical appraisal criteria for each eligible article (Supplementary Table S3). During the process of screening articles for eligibility, any disagreements regarding the critical appraisal were resolved through discussion among the assigned reviewers.

### Data synthesis and analysis procedures

After cleaning and modifying the extracted data on a Microsoft Excel spreadsheet, we employed STATA version 17 for further descriptive statistics, fixed effects regression, and random-effect regression to present the results of pooled TB-associated deaths for PLHIV (23). The *I*^2^ statistics and Cochran’s Q test were utilized to detect the degree of heterogeneity and elaborate on heterogeneity among studies, respectively (24). The source of heterogeneity among the included studies was further examined using the subgroup analysis and sensitivity test (23). The estimated risk factors obtained from each eligible study were combined and determined as a single estimated risk factor after performing a random effect meta-analysis regression analysis for TB-associated mortality among PLHIV in Ethiopia (23).

### Publication of biases and sensitivity analysis

The publication biases were assessed by inspecting funnel plots of the shape of the graph and quantitatively using Egger’s weighted regression test of a *p* value of <0.1 (23, 25, 26). The subgroup analysis was also conducted based on predefined themes such as study facilities (health center and hospital setup, study regions, and study population) for identifying further sources of heterogeneity and final policy implication based on the findings.

## Results

### Study screening process

**Figure 1:**
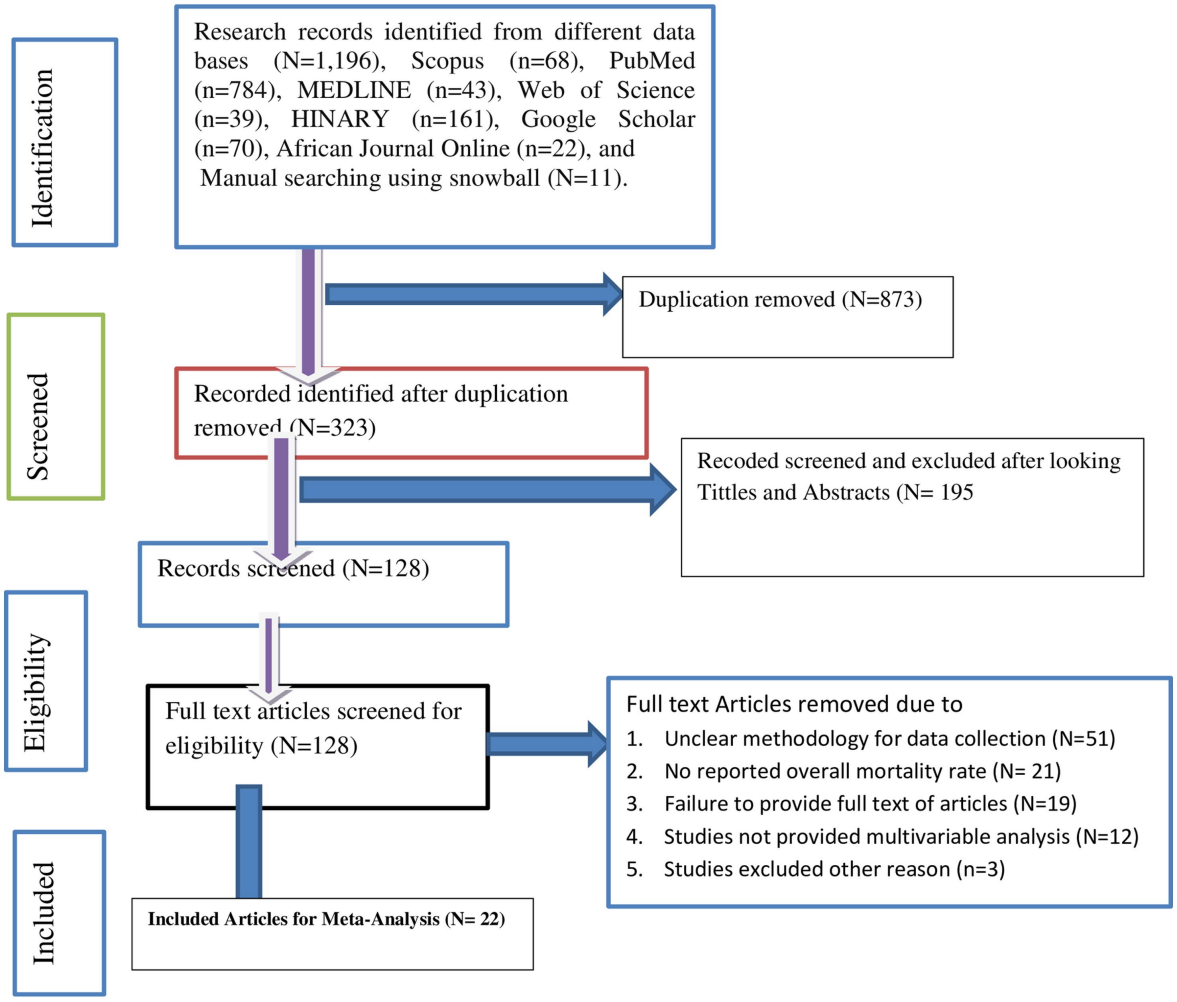
PRISMA flow diagram for articles searching diagram.

A total of 1,196 published articles were extracted from seven international electronic databases: Scopus (*N* = 68), PubMed (*N* = 784), MEDLINE (*N* = 43), Web of Science (*N* = 39), HINARY (*N* = 161), Google Scholar (*N* = 70), and African Journal Online (*N* = 22). Additionally, manual searching was employed, resulting in the identification of 11 articles using snowball techniques. After removing 873 duplicates, we assessed 323 articles. Among these articles, 195 were excluded based on titles and abstracts. We then screened the full text of the remaining 128 articles and excluded 51 due to unclear methodology, 21 for not reporting the overall mortality rate, 19 because the full text was inaccessible, 12 for not having reported multivariable analysis, and three articles for other reasons. Finally, 22 individual studies met the inclusion criteria for the meta-analysis shown in the PRISMA flow diagrams (5, 7, 27–44) (Figure 1).

#### Description reports of included studies

The included studies were extracted from six Ethiopian regions. Accordingly, five were from Amhara, five were from SNNR, three were from Oromia, three were from Tigray, three were from Harari, two were from Addis Ababa, and one was from a national-level (5, 7, 27–44) study. The smallest sample size was 169 from the Amhara region (28), and the largest number of participants was 1,123 from Addis Ababa (38).

Among the included studies, only nine (40.1%) were conducted on the adult population, whereas the remaining 8 (36.6%) and 5 (22.8%) studies involved children and both adult and children populations, respectively. More than half of the included studies, i.e., 81.5%, employed cohort study designs, and the remaining used cross-sectional designs with the study period ranging from 1 year (42) to 12 years (28) (Table 1).

**Table 1:** Characteristics of included articles for TB associated death for PLHIV in Ethiopia.

Name of author	Study year	Study region	Study design	Study population	Study setting	Quality	Sample size	Number of TB associated death	Study periods	Follow up time	Proportions of death
Sime et al. (7)	2022	Harari	RC	Both	HT	3	566	76	2014–2018	4 years	0.134
Shewano et al. (44)	2012	SNNR	RC	Adult	HC	3	370	50	2006–2010	6 years	0.135
Tolla et al. (40)	2019	Harari	RC	Both	HT	2	349	27	2012–2017	5 years	0.077
Belayneh et al. (30)	2014	Tigray	CS	Adult	HT	3	342	88	2009–2011	2 years	0.257
Gemechuet et al. (34)	2022	SNNR	RC	Children	HT	3	284	35	2009–2019	10 years	0.123
Haile Abrha et al. (35)	2015	Oromia	RC	Adult	HT	3	272	55	2010–2012	2 years	0.202
Esayas et al. (37)	2022	Oromia	RC	Adult	HT	3	402	84	2014–2022	8 years	0.209
Refera et al. (42)	2013	Oromia	RC	Adult	HT	2	501	79	2011–2012	1 years	0.158
Birhan et al. (31)	2021	Amhara	RC	Both	HT	3	243	87	2014–2019	5 years	0.358
Eshete et al. (33)	2022	Amhara	RC	Both	HT	3	407	74	2005–2015	10 years	0.182
Weldegebreal et al. (41)	2018	Harari	CS	Both	HT	3	627	54	2008–2014	6 years	0.086
Sileshi et al. (29)	2013	Amhara	RC	Both	HT	3	422	88	2009–2012	3 years	0.209
Emaby Gezae (32)	2019	Tigray	RC	Adult	HT	3	305	50	2009–2016	7 years	0.164
Ahmed et al. (27)	2016	AA	CS	Both	HT&HC	2	169	20	2012–2013	1 years	0.118
Teshome et al. [78]	2017	SNNR	CS	Adult	HT	3	188	24	2012–2015	3 years	0.128
Dawit et al. (21)	2021	SNNR	RC	Children	HT	3	274	47	2009–2018	9 years	0.172
Alula et al. (39)	2017	National	PC	Both	HT	3	355	55	2005–2016	11 years	0.155
Chanie et al. (5)	2021	Amhara	RC	Children	HT	2	227	39	2014–2021	7 years	0.172
Wondimu et al. (43)	2020	SNNR	RC	Adult	HT	3	364	83	2007–2017	10 years	0.228
Nigussie et al. (36)	2021	Tigray	RC	Children	HT	3	253	38	2008–2018	10 Years	0.150
Seyoum et al. (38)	2022	AA	RC	Adult	HT	3	1,123	50	2011–2018	7 years	0.045
Attale et al. (28)	2018	Amhara	RC	Children	HT	3	271	39	2005–2017	12 years	0.149

AA, Addis Ababa; CS, Cross sectional study design; ID, Incident proportions; RC, Retrospective cohort; SNNR, South nation nationalities region; HT, Hospital; HC, Health center; CPT, Cotrimoxazole preventive therapy; IPT, Isoniazid preventive therapy; PLHIV, People living with HIV/AIDS; TB, Tuberculosis.

### Tuberculosis associated mortality

**Figure 2:**
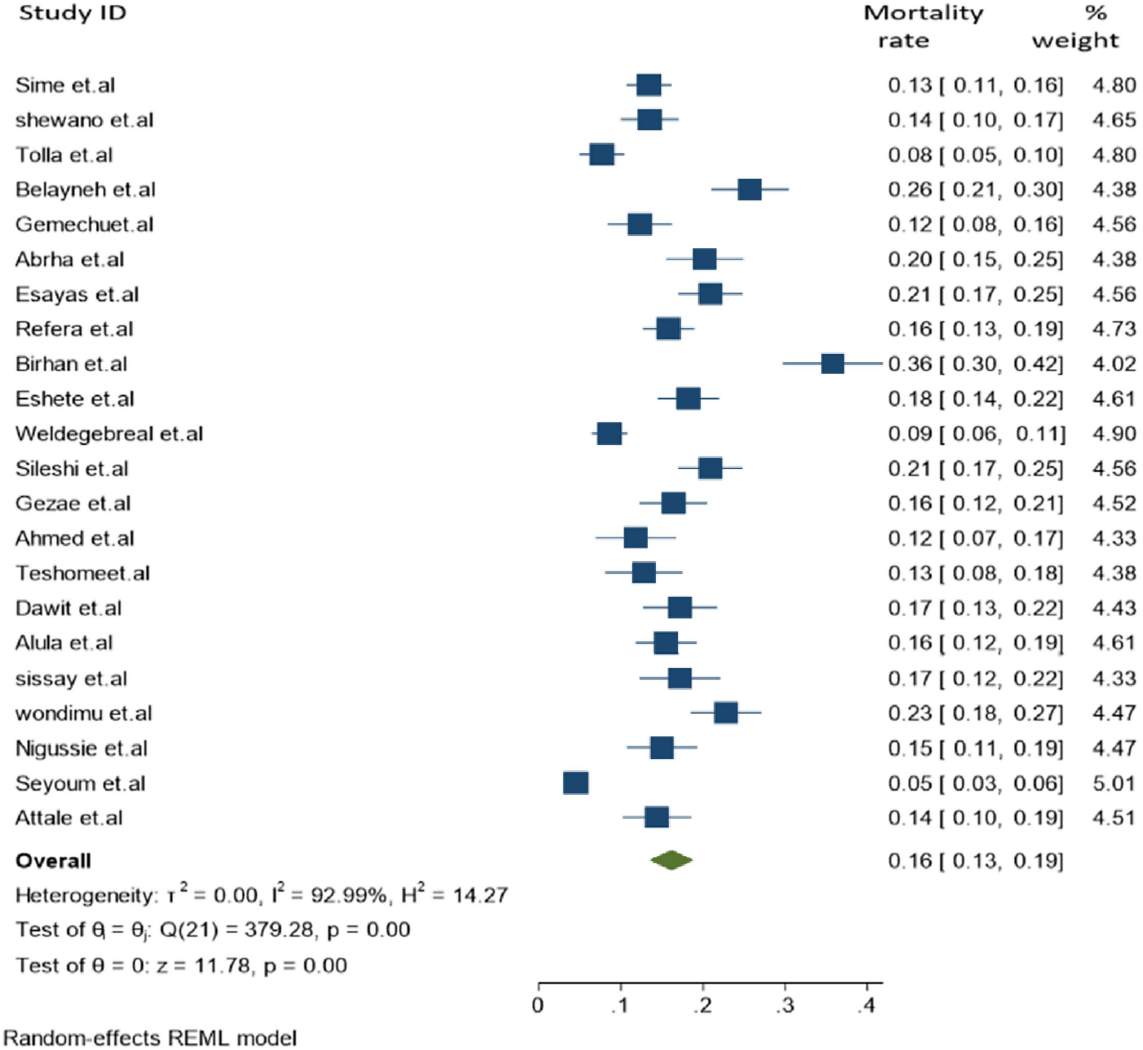
Forest plot for the polled tuberculosis-associated death of PLHIV in Ethiopia.

In the final meta-analysis, a total of 22 individual studies were included and a total of 9,856 cases of TB and HIV co-infection were screened, which reported 1,296 co-infected deaths. The overall pooled proportion of TB-associated mortality among people living with HIV (PLHIV) was found to be 16.2% [95% CI: 13.0–19.2, (*I*^2^ = 92.99%, *p* = 0.001)] (Figure 2).

### Subgroup analysis

**Figure 3:**
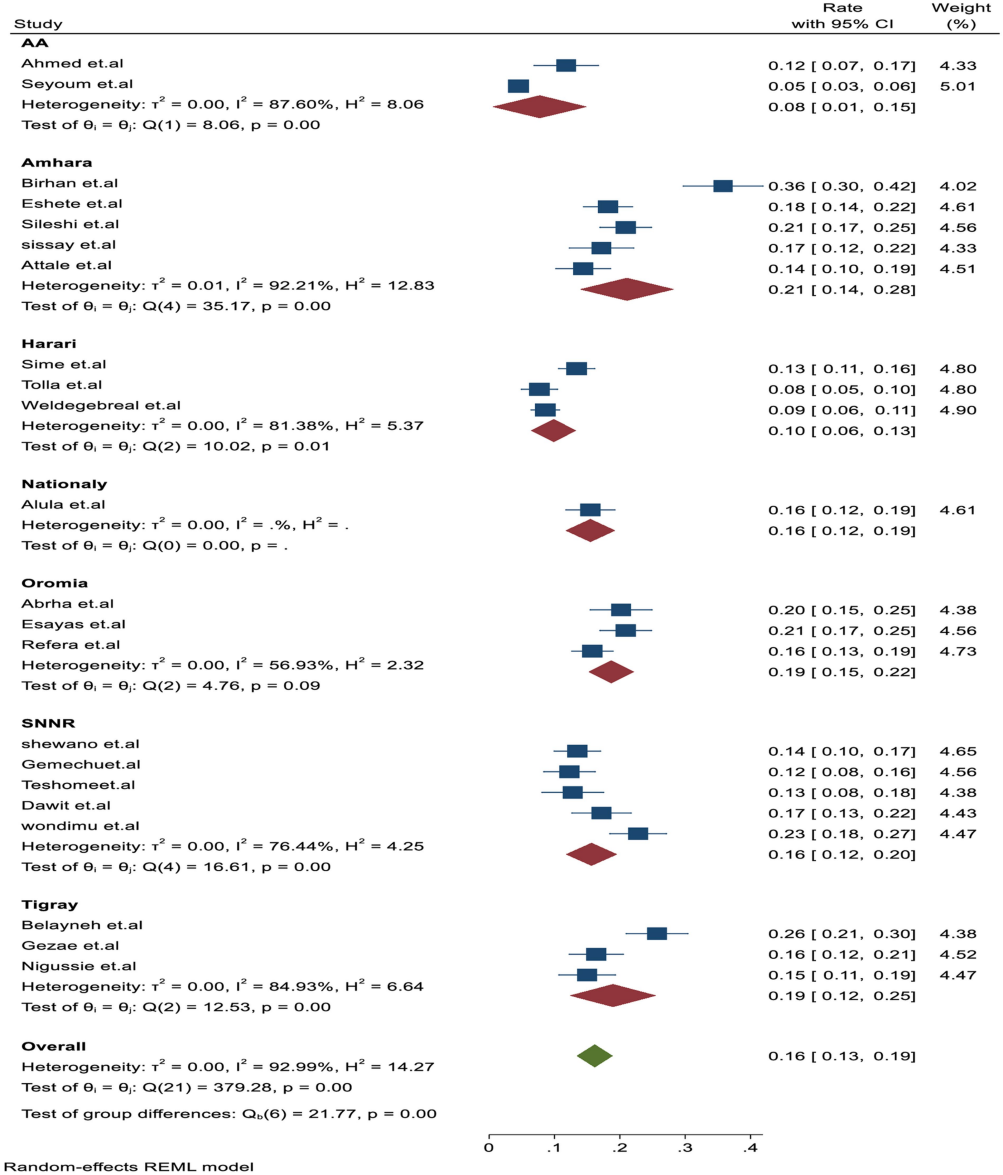
Subgroup analysis by study regions for tuberculosis-associated death of PLHIV in Ethiopia.

In the subgroup analysis, TB-associated mortality rates varied across each region, with the estimated rates being 21.1% (95% CI: 18.1–28.0, *I*^2^ = 84.4%) in Amhara, followed 19% (95% CI: 15–22, *I*^2^ = 56.95%) in Oromia, 19% (95% CI: 12.01–25, *I*^2^ = 6.64%) in Tigray, 16% (95% CI: 12.01–20, *I*^2^ = 76.44%) in SNNR, and 10% (95% CI: 6–13, *I*^2^ = 81.6%) in Harari, which had the lowest mortality rate compared to other regions (Figure 3).

**Figure 4:**
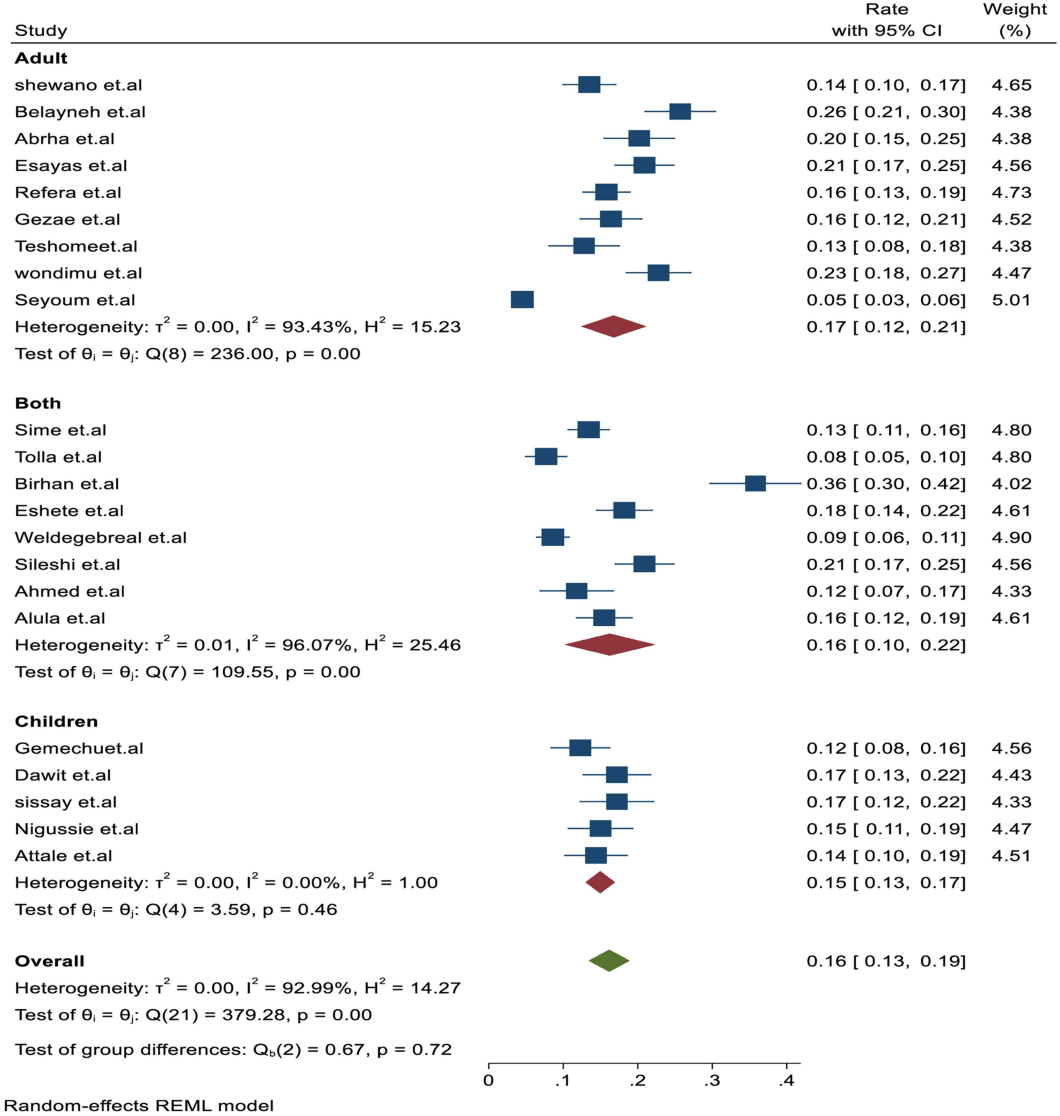
Subgroup analysis by the study population for tuberculosis-associated death for PLHIV.

Similarly, the subgroup analysis based on the study subjects was higher in adults with 17% (95% CI: 12–21.01, *I*^2^ = 93.4%) than in studies that included both adult and children participants with 16% (95% CI: 10–20.01, *I*^2^ = 96.4%) and children alone with 15% (95% CI: 13–17, *I*^2^ = 0.001%), respectively (Figure 4). In addition, based on the study setting, a higher proportion of TB-associated mortality of 17% (95% CI: 14–19, *I*^2^ = 93.8%) was observed among studies conducted in hospital settings compared to those conducted in health centers, which reported 14% (95% CI: 10.0–17.01, *I*^2^ = 0.001), and in hospital setups, which reported 12% (95% CI: 7–17, *I*^2^ = 0.001%).

#### Publication bias

**Figure 5:**
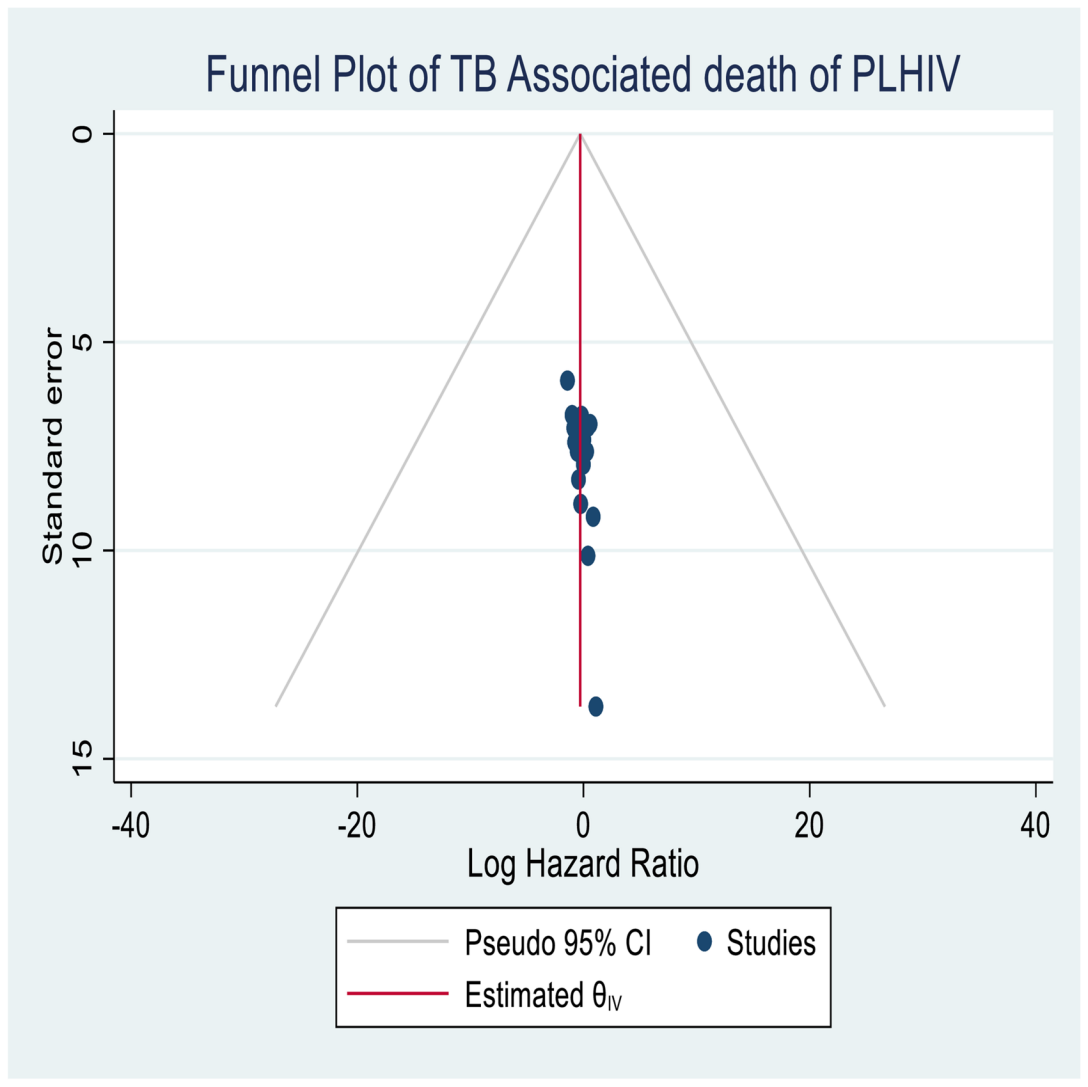
Publication bias assessment for TB-associated mortality of PLHIV in Ethiopia.

The publication bias was assessed using funnel plots, as well as quantitative methods such as Begg’s and Egger’s tests (45, 46). The final publication result indicated no evidence of publication bias, and all studies were included in the plots, suggesting no significant publication biases in both graphical and quantitative estimation (Figure 5).

### Sensitivity analysis

The sensitivity analysis showed that all studies were within the confidence interval bounds of the meta-regression, as shown in Egger’s (*p* = 0.01) and Begg’s tests (*p* = 0.01) with no significant publication bias.

#### Predictors for TB-associated death

In this systematic review, we categorized adjusted odds ratios from primary studies into themes to identify the risk factors for tuberculosis (TB)-associated mortality of PLHIV. Thus, TB-associated mortality was assessed mainly in four categorical themes, including WHO advanced clinical stages (III and IV), declining CD4 count (≤200 cell/mm^2^), missed IPT, and missed CPT. Accordingly, missed IPT, missed CPT, and WHO advanced clinical stages (III and IV) themes were significantly associated with mortality during co-infection (Table 2).

**Table 2:** Random effect meta-regression of factors associated with TB-associated death of PLHIV in Ethiopia.

Variables	No. of studies	Population	AHR	95%CI	*I*^2^	Tau^2^	*p* value
Advanced WHO Clinical stages, Stages I&II	11	3,737	Ref	Ref	Ref	Ref	Ref
Stage III&IV			3.01	[1.9–4.7]	89.5%	0.45	0.001^*^
Cotrimoxazole preventive therapy (CPT) given	7	2,229	Ref	Ref	Ref	Ref	Ref
Not given		1.89	2.56	[1.05–2.85]	93.0%	0.56	0.03^*^
Isoniazid preventive therapy status (IPT) given	4	1,200	Ref	Ref	Ref	Ref	Ref
Not given			1.8	[1.46–2.31]	96.6%	0.001	0.001^*^

#### Effects of advanced clinical stages on TB-associated death

**Figure 6:**
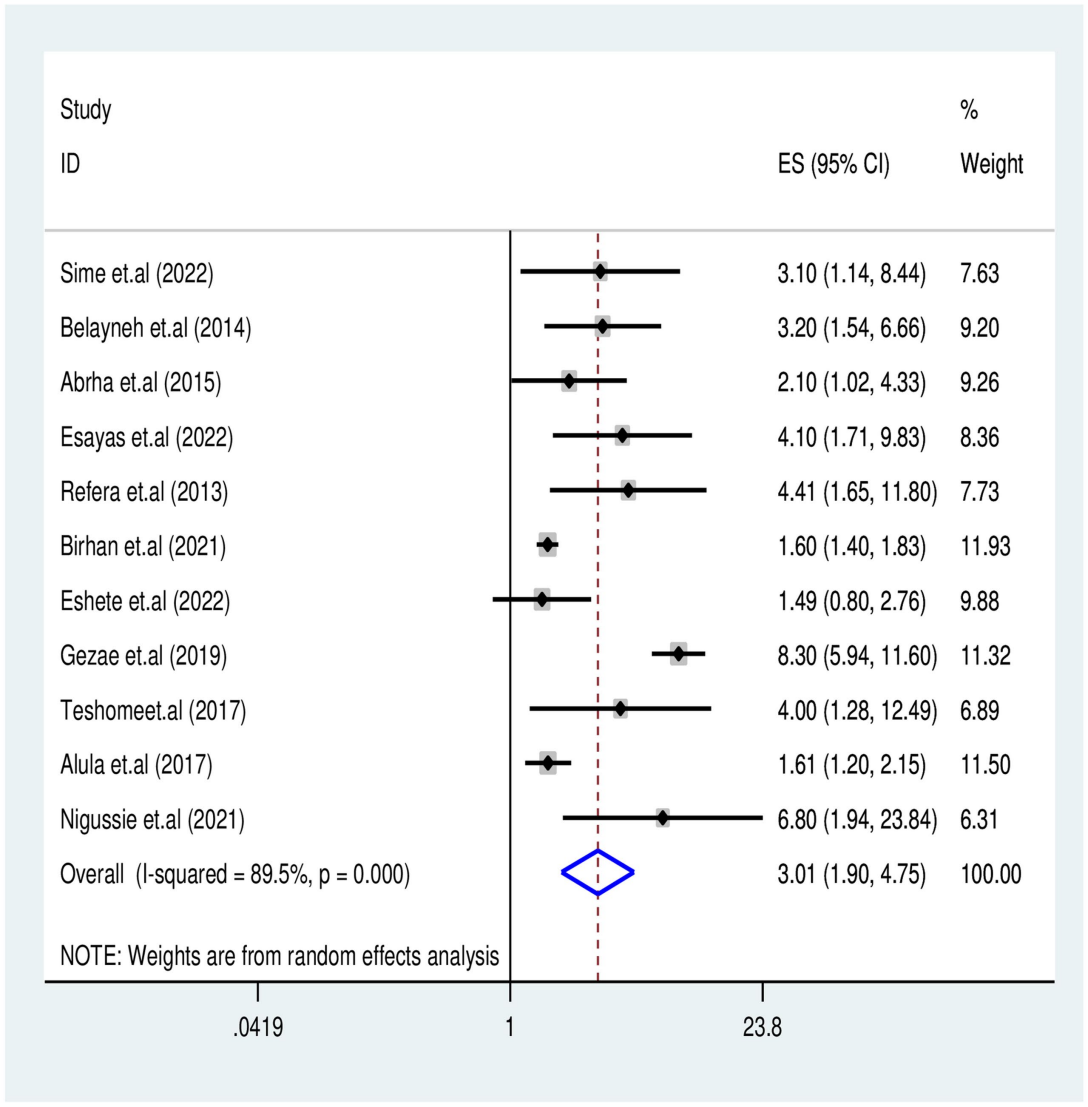
Association of WHO Clinical Stages III&IV with TB associated mortality for PLHIV in Ethiopia.

We analyzed 11 studies (7, 20, 30, 31, 33, 36, 37, 39, 42, 47, 48) that examined the association between WHO clinical stages (III and IV) and TB-associated mortality among 3,737 participants. The results indicated that participants in the advanced WHO clinical stages III and IV had an increased risk of TB-associated death compared with their counter groups [OR = 3.01 (95% CI: 1.9–4.7)] with (Tau2 = 0.45, *I*^2^ = 89.5%, *p* = 0.001) (Figure 6).

#### Effects of missed IPT on TB-associated death

**Figure 7:**
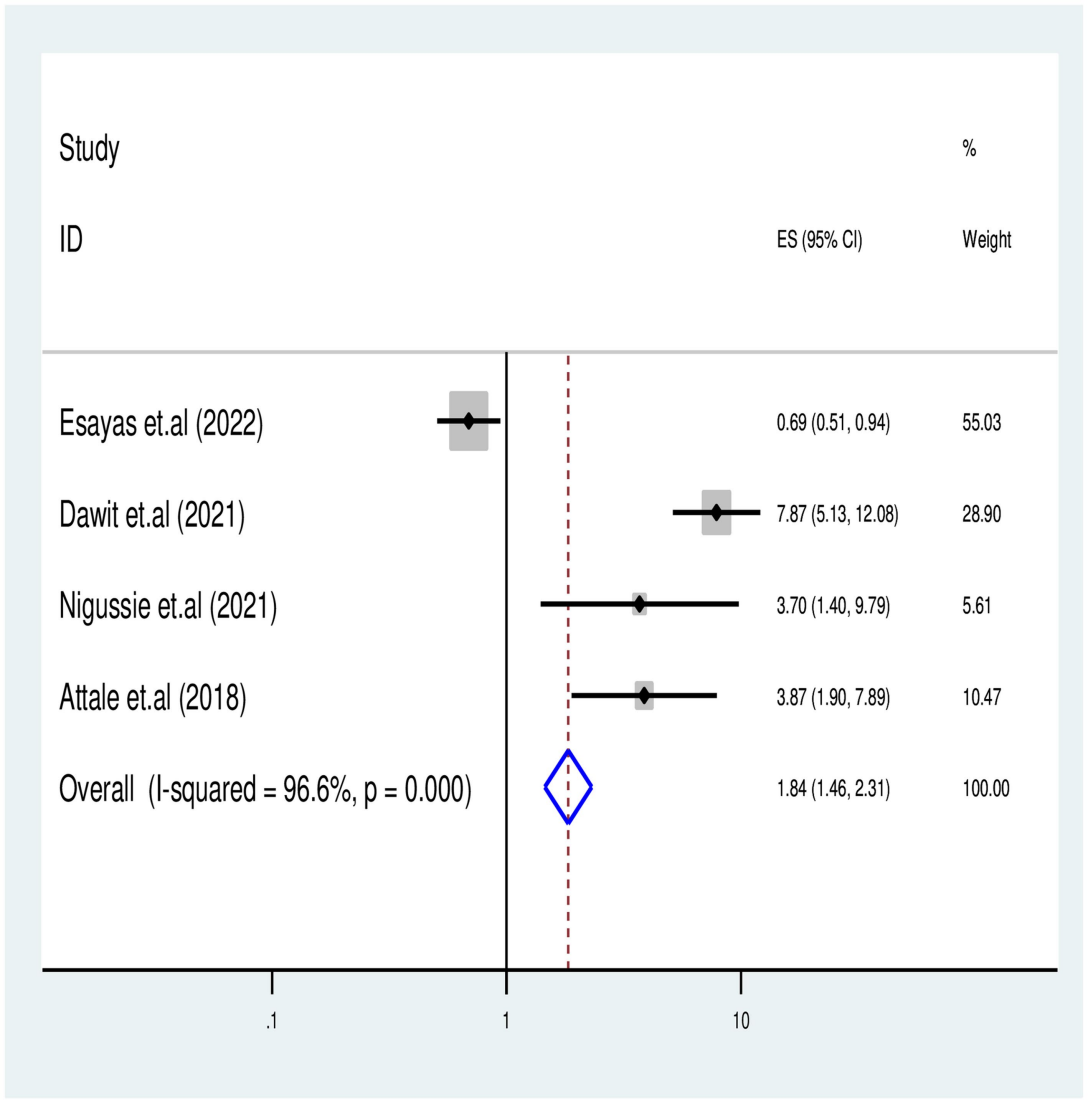
Association between missed IPT and TB associated death for PLHIV in Ethiopia.

We identified four published articles investigating the association between missed isoniazid preventive therapy (IPT) and TB-associated death. Three of these studies reported a significant risk of TB-associated death with missing IPT, while one study found no significant association. A meta-regression analysis involved 1,200 study participants, and the meta-analysis revealed that PLHIV who missed IPT had a 2-fold risk of TB-associated death compared to those who received IPT [OR = 1.8 (95% CI: 1.46–2.31) with (*I*^2^ = 96.6%, *p* = 0.001)] (Figure 7).

#### Effects of missed CPT on TB-associated death

**Figure 8:**
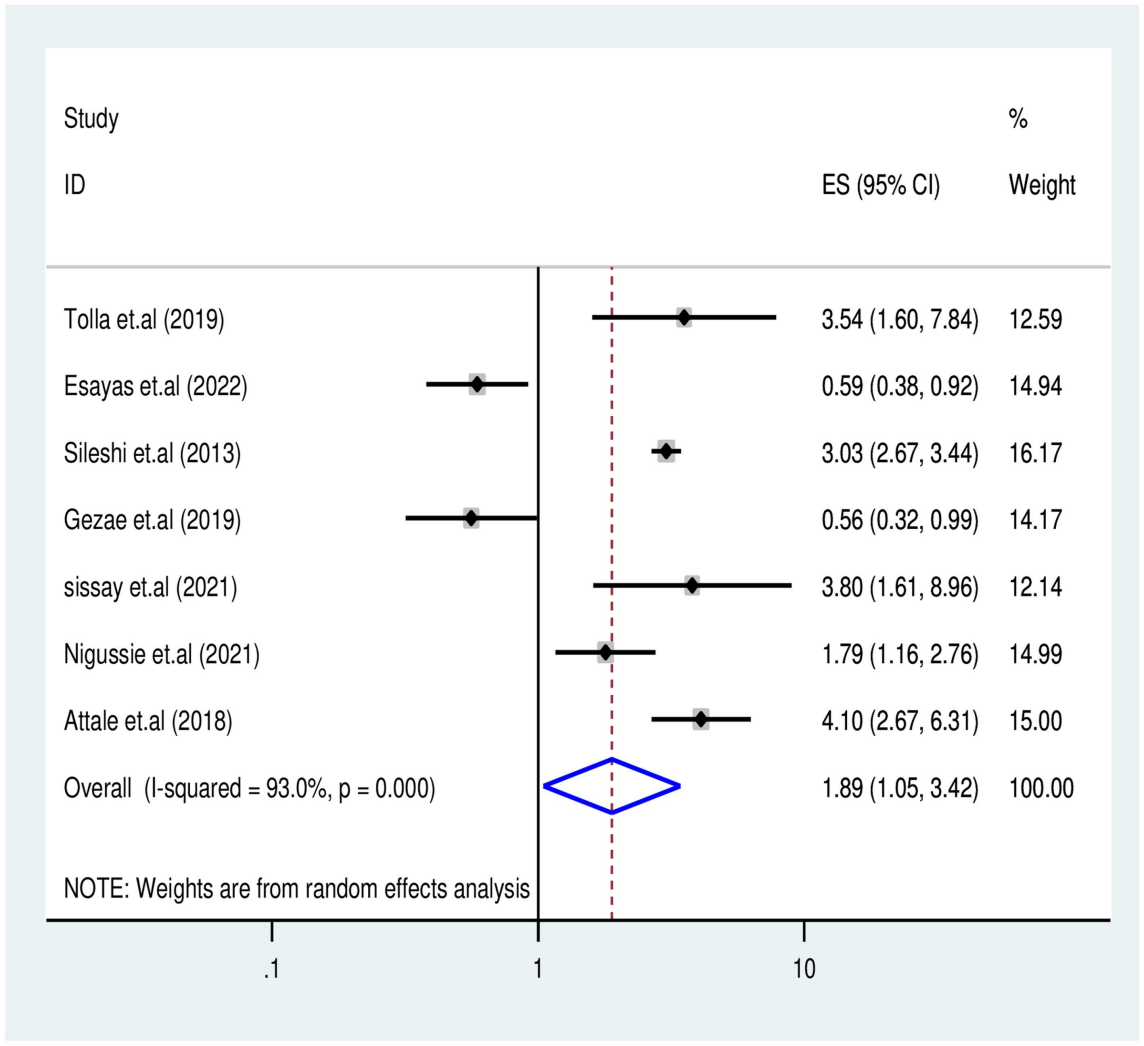
Association between missed CPT and TB associated death for PLHIV in Ethiopia.

The association between missed CPT and TB-associated death was examined in five studies (5, 20, 29, 36, 37, 40) involving 2,229 participants. Five studies reported a significant association with missed CPT, and the final meta-regression analysis revealed that missed CPT doubled (OR: 1.89, 95% CI: 1.05–3.42) the risk of mortality (*I*^2^ = 93%, *p* = 0.035) (Figure 8).

#### Effects of declining CD4 count on TB-associated death

A total of six studies (7, 30, 37, 40, 42, 43) investigated the impact of declining CD4 count on TB-associated mortality among PLHIV. The final meta-regression analysis revealed no significant association between CD4 count ≤200 cells/mL and increased mortality risk (OR = 2.18, 95% CI: 0.97–3.08; *I*^2^ = 82.8%, *p* = 0.064).

## Discussion

In the final report of this systematic review and meta-analysis, we included 22 published studies, encompassing a total of 9,856 cases of TB and HIV co-infection. Among these cases, a total of 1,296 deaths were reported. The highest mortality was observed in the Tigray region (30) and the lowest mortality in Addis Ababa city (27). The overall pooled proportion report of TB-associated death in Ethiopia was estimated at 16.2% (95% CI: 13.0–19.2) (*I*^2^ = 92.99%, *p* = 0.001). This finding is significantly higher than the previous meta-analysis that reported 4.3% in South Africa (19), 9.2% in Amsterdam (49), and 11.1% in England (50), and 2.8% in the Netherlands (51). The possible reason for the estimated difference is poor screening and diagnosis of TB by healthcare providers during successive follow-ups for PLHIV, poor adherence to treatment guidelines, low completion rate for IPT among patients during successive follow-ups, and reports of higher mortality compared with the developed nations (3). Conversely, the estimated proportions of TB-associated mortality are lower than those reported elsewhere in systematic review reports: 17.7% in Geneva (6) and 40.1% in London (52). This difference may be attributed to differences in the included studies and recruitment study periods. Furthermore, the possible reason for the estimated difference between the two countries might be the effect of poor screening and diagnostic ability of TB by healthcare providers among suspected PLHIV in Ethiopia compared to the highly sophisticated diagnosing equipment for TB detection available in countries such as Geneva and London.

In this report, the observed heterogeneity among studies during the meta-analysis report was assessed by using a subgroup analysis, including predetermined themes of study region, population, and follow-up time. Accordingly, the Amhara region had the highest proportion of TB-associated mortality at 21.1% (95% CI: 18.1–28.0, *I*^2^ = 84.4%, *p* = 0.001) compared to Addis Ababa, which had 8% (95% CI: 1.1–15, *I*^2^ = 87.6%, *p* = 0.001). This difference may be attributed to the ongoing civil war in the Amhara region since July 2021, leading to the loss of life and displacement of more than 1.4 million population who ended up living in slums and destitution, including PLHIV, where, tuberculosis fatality and transmission dynamics are high (53, 54).

In this review, factors associated with TB-associated mortality for PLHIV were identified from aggregated studies; patients who missed CPT experienced a two-fold increase in mortality risk compared to those who received CPT during follow-up periods (AHR = 1.89, 95% CI: 1.05–3.42, *I*^2^ = 93%, *p* = 0.03). This finding is consistent with the findings of previous primary studies (3, 5, 20, 29, 36, 37, 40) and meta-analyses reported by universities in Gondar (55) and Debre Markos (56). This consistency in findings may be attributed to cotrimoxazole, which is an effective drug against the incidence of lethal opportunistic infections, including TB, which can increase immunosuppression and mortality risk.

This systematic review found that PLHIV co-infected with TB and in advanced WHO clinical stages (III and IV) had 3.01 times increased risk of death compared to those in stages I and II (AHR = 3.01, 95% CI: 1.9–4.7). This finding is consistent with previous reports from universities in Debre Markos (57), Gondar (10), and Addis Ababa (58). This association might be related to PLHIV who developed TB commonly at the advanced WHO clinic stages, leading to a higher risk of having a reduction in CD4 count and decreased cellular immunity. This decline accelerated the existence of rapid viral replication and indirectly contributed to the increased likelihood of premature mortality among PLHIV.

The final report of this meta-analysis revealed that PLHIV who missed IPT had a significant risk of mortality during TB co-infection disease courses. Accordingly, TB-associated mortality showed a two-fold increase for those who missed IPT as compared to those who completed IPT during successive follow-ups of care (AHR = 1.8: 95% CI 1.46–2.32). This finding aligns with the findings of a previous meta-analysis conducted in Ethiopia (10, 57) and is supported by previous premier studies (59–61). In line with the above finding, the concomitant use of IPT with ART administration has been shown to reduce or demote new cases of TB incidence and associated deaths by over 90% among PLHIV (10). However, factors such as IRIS, inadequate screening by a caregiver, low IPT completion, and poor comprehensive care during successive follow-ups have been associated with poor treatment outcomes.

In contrast to previous systematic review findings (6, 14, 15, 52, 55) and primary studies (6, 7, 14, 15, 30, 37, 40, 42, 43, 52, 55), this meta-regression found no significant association between declined CD4 count (≤200 cells/mL), age of patients, duration of follow-up, comorbidity status, and body mass index with the risk of death during TB and HIV co-infection treatment (AHR = 2.18, 95% CI: 0.97–3.08, *I*^2^ = 82.8%, *p* = 0.064). This lack of association might be related to the methodological differences, heterogeneity of included studies and populations, sample size limitations, publication bias, and unaccounted factors, and further experimental studies are highly needed to understand this relationship better.

### Strengths and limitations of the study

The strengths of this study included a comprehensive search and involvement of multiple authors. In addition, it included 22 studies with 8,315 TB-HIV co-infected cases, providing valuable mortality screening data for national-level representation. However, it is important to consider the limitation that the majority of the studies employed a retrospective nature of data collection and thus were limited to specific regions in Ethiopia, which may impact the generalizability of the results.

### Conclusion and recommendations

In Ethiopia, the mortality rate among individuals co-infected with TB and HIV is notably high, with nearly one-fifth of individuals (16%) succumbing during co-infection, which is higher compared to other African countries. Risk factors for death during co-infection were identified; the included studies examined advanced WHO clinical stages IV and III, hemoglobin levels (≤10 mg/dL), missed isoniazid preventive therapy (IPT), and missed cotrimoxazole preventive therapy (CPT) as predictors. To reduce premature deaths, healthcare providers must prioritize active TB screening, ensure timely diagnosis, and provide nutritional counseling in each consecutive visit.

## Data availability statement

The original contributions presented in the study are included in the article/Supplementary material; further inquiries can be directed to the corresponding author.

## Author contributions

FKB: Conceptualization, Data curation, Formal analysis, Funding acquisition, Investigation, Methodology, Project administration, Resources, Software, Supervision, Validation, Visualization, Writing – original draft, Writing – review & editing. TKB: Data curation, Formal analysis, Funding acquisition, Methodology, Project administration, Resources, Supervision, Validation, Visualization, Writing – review & editing. SAM: Formal analysis, Project administration, Software, Visualization, Writing – original draft, Writing – review & editing. AAK: Formal analysis, Funding acquisition, Project administration, Resources, Supervision, Validation, Visualization, Writing – original draft, Writing – review & editing. MWA: Conceptualization, Funding acquisition, Investigation, Resources, Software, Writing – original draft, Writing – review & editing. NS: Data curation, Funding acquisition, Methodology, Resources, Supervision, Visualization, Writing – original draft, Writing – review & editing. BBA: Conceptualization, Data curation, Formal analysis, Funding acquisition, Investigation, Resources, Software, Supervision, Validation, Visualization, Writing – original draft.

## Supplementary material

SUPPLEMENTARY TABLE S1 PRISMA CHEKLIST 2020.

SUPPLEMENTARY TABLE S2 Final meta dataset.

SUPPLEMENTARY TABLE S3 JBI critical appraisal checklists.
