# Supplementary material for: Tuberculosis-associated mortality and risk factors for HIV-infected population in Ethiopia: a systematic review and meta-analysis
Source: Front Public Health. 2024 Jul 22;12:1386113. doi: 10.3389/fpubh.2024.1386113 (PMC11298472; doi:10.3389/fpubh.2024.1386113)
Supplement: SUPPLEMENTARY TABLE S1 — PRISMA CHEKLIST 2020. [file Table_1.doc]

| **Section/topic** | **#** | **Checklist item** | **Reported on page #** |
| --- | --- | --- | --- |
| **TITLE** | | |  |
| Title | 1 | **Tuberculosis-associated mortality and risk factors for HIV infected population in Ethiopia; Systematic review and meta-analysis** | 1 |
| **ABSTRACT** | | |  |
| Structured summary | 2 | **Abstract**  **Background;** Despite the effectiveness of antiretroviral treatment (ART) in reducing deaths from opportunistic infections among people living with HIV (PLHIV), Tuberculosis (TB) remains a significant cause of mortality, accounting for one in every three deaths in this population. However, in Ethiopia, there is a lack of aggregated and comprehensive data on the disease progression and treatment outcomes during the co-infection phases. This meta-analysis report is aimed to estimate the pooled proportion of Tuberculosis-associated mortality and predictors for people living with HIV in Ethiopia  **Methods:** A comprehensive review and analysis were conducted without time restriction. The study adhered to PRISMA guidelines and assessed article quality using the JBI checklist. Pooled estimates were calculated using the random-effects model regression in STATA version 17. The heterogeneity of the studies was evaluated using the Q test and I2 statistic. Subgroup analysis, publication bias assessment, and sensitivity analysis were also conducted. This systematic review and meta-analyze are registered in Prospero with CRD42024509131  **Results:** Over all 22 individual studies were included in the final meta-analysis reports. During the screening of 8,315 co-infected cases of TB and HIV, 1242 deaths were reported. The Amhara and Tigray regions had the highest number of co-infection death of cases were reported (88/422) and 88/342), while Addis Ababa had lowest number of (20/169) co-infection death of cases was reported. The final meta-analysis estimation of the pooled TB-associated mortality in Ethiopia was found to be 16.2% (95% CI: 13.0-19.2) with significant heterogeneity among studies (I2=92.9%, P=0.001). In the subgroup analysis, the Amhara region had a high TB-associated mortality rate observed region with 21.1% (95% CI: 18.1-28.0, I2=84.4%, P=0.001) compared with Addis Ababa, 8% (95% CI: 1.1-15, I2=87.6%, P=0.001) and Harari region 10% (95%CI: 6% - 13.1%, I2=83.38%,P=0.001). TB-related death of PLHIV was significantly associated during random effect met-regression with WHO clinical stages III&IV (OR=3.01, 95% CI: 1.9-4.7, P=0.001), missed co-trimoxazole preventive therapy (CPT) (OR=1.89, 95% CI: 1.05-3.42, P=0.03), and missed isoniazid preventive therapy (IPT) (OR=1.8, 95% CI: 1.46-2.3, P=0.001).  **Conclusion;** In this meta-analysis reports, nearly one in every five (20%) individuals with TB and HIV co-infected have died in Ethiopia. Healthcare providers should prioritize active TB screening, timely diagnosis, and treatment to reduce premature mortality, alongside enhancing adherence counseling with uninterrupted ART provision is needed to demote premature death. | 1 |
| **INTRODUCTION** | | |  |
| Rationale | 3 | **Introduction**  People living with Human Immune deficiency virus (PLHIV) are more susceptible to tuberculosis (TB), which is a leading cause of mortality(1, 2). There is a strong synergy between HIV infection and tuberculosis, while people living with HIV (PLHIV) are at high risk of dying from tuberculosis (TB) and HIV infection is the biggest risk factor for active TB by declining the cellular immunology and increased latent TB reactivation in the lungs(3, 4).  Globally in 2020, there were 10 million new TB cases, 1.2 million TB deaths occurred in HIV-negative people, and 208,000 in HIV-positive individuals(5). The TB incidence in sub-Saharan Africa is high at 2,017 cases/100,000 patient-years. Pre- and post-antiretroviral therapy incidence was 5.4-10.5 cases/100 person-years with 0.3 million deaths worldwide in 2017 and left lifetime risk 15-22%(6). Despite widespread treatment and breakthroughs in detection, TB-associated case mortality accounted for 25%–40% of global deaths and 18%–25% of PLHIV admissions(7-9). The WHO global TB study estimates that in 2021, there will be 42 HIV-positive tuberculosis incidence rates and 212 TB occurrences per 100,000 people in Africa(10).  Tuberculosis (TB) remains a leading cause of HIV-associated mortality and morbidity for adults and children worldwide(11). Previous systematic findings reported that PLHIV and TB are associated with 37% of deaths in sub-Saharan African countries with a substantial contribution to hospitalization (11, 12). In Africa, an estimated 1.5 million deaths were recorded from TB infection, where the co-infection death epidemic contributed 214,000 (14.30%) of all recorded in 2021(1). In sub-Saharan African countries 10%–15% co-infection mortality burden with more than 51% lifetime risk(1, 2). Ethiopia was among 30 TB and HIV high-burden countries in 2016 with 36,761 new HIV cases for ART with 5.9% active TB co-incidence(1, 13).  Several previous studies' findings suggested (6, 9, 10, 14-17) including the meta-analysis reported(11, 18-20) PLHIV co-infected with TB patients in the era of ART patients, being BMI ≤18.5 kg/m2, WHO advanced clinical stage (III&IV), alcohol consumption, missed IPT, poor ART adherence, missed CPT and treatment failure were significantly caused for TB associated death for PLHIV.  In Ethiopia, deaths with the twine epidemics of TB and HIV varied across each region with 23.01 cases per 100 person-years in Tigray (21) for adults and 17.15 cases per 100 years for children in the south nation and nationalities region (22). In some instances, children living with HIV face disproportionate deaths reported from the epidemics since their young age category and immature immunity making them highly risky(4, 23). However, concurrent administering of IPT with highly active antiretroviral therapy (HAART) after ruling out active TB symptoms demoted more than 90% of TB-associated incidence of death (3, 24, 25). Despite this evidence of the effectiveness of HAART in reducing mortality for PLHIV, there is limited national-level co-infection mortality of aggregated data for PLHIV. Therefore, this systemic review and meta-analysis aimed to estimate the proportion of TB-associated mortality for PLHIV in Ethiopia | 4 |
| Objectives | 4 | The objective of this meta-analysis is/are   - To estimate the pooled Tuberculosis-associated mortality of PLHIV in Ethiopia? - To identified predictors for TB-associated death for PLHIV post-ART? | 4 |
| **METHODS** | | |  |
| Protocol | 5 | Based on the information provided, the protocol of the study can be accessed through the web address ([https://www.crd.york.ac.uk/prospero/#recordDetails](https://www.crd.york.ac.uk/prospero/#recordDetails)). The protocol registration number for the study is **CRD42024509131** | 4 |
| Eligibility criteria | 6 | We included scientific papers that reported the following criteria for the final analysis.   1. We included studies that reported TB and HIV co-infected mortality in Ethiopia. 2. We included observational studies (cross-sectional and cohort studies) conducted either published or unpublished and were selected for the final meta-analysis. 3. The studies were included regardless of diagnostic methods, all participants with HIV positive patients and started HIV/AIDS care from December 30, 2012, to January 1^st^, 2023. 4. Articles containing the estimated results of prevalence, incidence, and /or mortality. 5. A scientific paper that was written in English version and published before the last date of the article was searched on November 30, 2023. | 5 |
| Information sources | 7 | We used mainly five databases both for published and unpublished articles such as PubMed, MEDLINE, HINARY, Africa Journals Online, and Google Scholar databases for searching for articles. Terms (MeSH) that are part of the controlled vocabulary and free text were taken from articles that qualified for the final report. | 5 |
| Search | 8 | Using the specified eligible criteria and the following MeSH terms: (1) mortality, (2) incidence, (3) tuberculosis, (4) HIV infection, (5) individuals, (6) children, (7) adults, and (8) Ethiopia, the included articles were extracted. The authors employed the following search terms to retrieve relevant studies from databases: "Epidemiology" OR "Incidence" AND "Death", OR "Mortality”, OR” Case fatality” AND "Tuberculosis", OR "Pulmonary Tuberculosis", OR "Disseminated Tuberculosis", OR "lymphadenitis”, AND "HIV", OR "AIDS”, AND " Children" OR "Pediatrics" OR “Infant” OR "Adult" AND “Ethiopia". The search process involved three authors (FK, DT & TK) selected the most pertinent studies based on predefined criteria**.** | 4 and 5 |
| Study selection | 9 | The Included articles in this study had cross-sectional, retrospective cohort, and prospective cohort study designs published from December 30, 2012, to November 30, 2023 | 5 |
| Data collection process | 10 | Four writers (FK, TK, BBA, and MW) independently extracted the data and evaluated the quality of each study by determining the eligibility of the titles and abstracts of the studies after removing duplicates. Discussion was used to settle any disagreement or uncertainty that arose during the article extraction process. These reviewers assessed the full-text articles; if one or more of them believed an article could be significant, it qualified. After the article was carefully examined for its titles, abstracts, and full text, four authors (FK, TK, NS, and BB) used a Microsoft Excel spreadsheet to extract the specifics of each article. Through discussion with a third-party reviewer (SA and MW), the disagreement during data extraction using the critical appraisal process was resolved to comply with the Preferred Reporting Items for Systematic Reviews and Meta-Analysis Protocols (PRISMA-2020) criteria[28]**.** Finally, all eligible studies were approved by all author's agreements and any differences were worked out through discussion with reach censuses. Following the agreement, information about principal investigators, year of publication, study period, study setting, study population, and sample size was retrieved from the identified articles. Each of the included studies’ the risk of bias was assessed by all listed authors (FK, TS, BB), evaluated, and screened. The Joanna Briggs Institute of Critical Appraisal (JBI) checklist was used to evaluate the papers' quality, and the results were incorporated into the final meta-analysis. In order to categorize the final report into three categories—good, bad, and fair quality results—we used a variety of checklists, depending on the study designs of the published papers. Any disagreements among reviewers regarding the critical appraisal were settled through discussion and consensus-building. | 6 |
| Data items | 11 | Previously published item published from December 30, 2012, to November 30, 2023 | 6 |
| Risk of bias in individual studies | 12 | The Higgs I^2^ statistics were also utilized to detect heterogeneity. Heterogeneity between studies was elaborated using Cochran's Q test and quantified with the I^2^ statistics[31]. The degrees of statistical heterogeneity between the studies were assessed using I2 statistics; values of 25%, 50%, and 75% were thought to indicate modest, medium, and high levels of heterogeneity, respectively [38]. The source heterogeneity among the included studies was further examined using the subgroup and sensitivity analyses. Given the limited number of studies, a P-value of ≥ 0.1 was deemed to indicate statistically significant heterogeneity[29]. For further clarification on the source of heterogeneity, the random effect meta-regression was reported on the study setting, regions, and study population done by subgroup analysis. In the final meta-analysis review, the estimated risk factors obtained from each study were pooled and determined as a single estimate with its corresponding 95% confidence interval. The random effect regression model was used for the data-identified heterogeneous analysis [29]. | 7 |
| Summary measures | 13 | The publication biases were assessed by visual inspection of funnel plots of the graph and quantitative using Egger’s weighted regression at p <0.1[32, 33]. In addition, we performed a leave-one-out sensitivity analysis to confirm a study with a biased direction of pooled estimates of Begs and Eggers tests[29]. | 7 |
| Synthesis of results | 14 |  | 7 |

Page 1 of 2

| **Section/topic** | **#** | **Checklist item** | **Reported on page #** |
| --- | --- | --- | --- |
| Risk of bias across studies | 15 | The publication biases were assessed using inspection of funnel plots of the shape of the graph, quantitatively using Egger's weighted regression test of the P-value <0.1[32, 33], and using a leave-one-out sensitivity analysis to confirm that there were no studies potentially biased direction using Begs and Eggers test[29]. | 7 |
| Additional analyses | 16 |  | 7 |
| **RESULTS** | | |  |
| Study selection | 17 | **Study screening process**  To extract articles, we used five databases: Africa Journals Online, Google Scholar, HINARY, MEDLINE, PubMed, and HINARY. 1196 main studies were found overall as a result of our thorough search. Out of these, two articles were found manually by looking through other articles' references. Following a comprehensive assessment, 1082 articles were determined to be duplicates and were removed from the analysis. After eliminating 82 publications that were duplicates, the final set of 22 articles that satisfied the meta-analysis's inclusion requirements was obtained [6, 8-10, 13, 16, 19, 20, 34-45] **(Figure 1).** | 7 |
| Study characteristics | 18 | The studies were mostly carried out in six Ethiopian regions. Thus, five of the studies came from Amhara[8, 9, 16, 35, 36], four were from SNNR [6, 10], and six papers were from the Oromia and Harari regions [19]. Whereas, the remaining five articles were from Tigray [38, 39] and Addis Ababa regions [20]. Regarding participant selection, the smallest study sample was 169 from Amhara [35], and the largest sample was 1123 from Addis Ababa [20] regions[35]. A total of 8315 cases of TB with HIV co-infection were screened, resulting in 937 TB-associated deaths were observed. Out of the included studies, 9 (40.1%) focused on adults, 8 (36.6%) on children, and the remaining 5 (22.8%) included both adults and children. Additionally, 18 studies were deemed of good quality, while 4 were considered fair. The majority (77%) of the studies were cohort studies, with the rest being cross-sectional and follow-up studies. The study period ranged from one year [43] to twelve years of follow-up [35] (Table 1). | 8 |
| Risk of bias within studies | 19 | **Using Subgroup analysis and funnel plot, sensitivity test was done to assessed risk of biases with in studies**  To address the considerable heterogeneity observed in the final meta-analysis, further subgroup analysis was conducted based on predetermined characteristics such as study region, population, and study design. As a result, the Amhara region exhibited the highest tuberculosis (TB) associated mortality among people living with HIV (PLHIV) at 21.1% (95% CI: 18.1-28.0) with significant heterogeneity (I2 = 84.4%, p < 0.001) compared to the SNNR region at 16% (95% CI: 12.01-20.0, I^2^ = 76.44%, p = 0.001), Addis Ababa at 8% (95% CI: 1.1-15, I^2^ = 87.6%, P = 0.001), Harari at 10% (95% CI: 6-13, I^2^ = 81.6%, P= 0.001), Oromia 19% (95% CI: 15--22, I^2^ = 56.95%, P= 0.001), and 19% (95% CI: 12.01-25, I^2^ = 6.64%, P=0.001**)** of Tigray regions. | 13 |
| Results of individual studies | 20 | **Pooled Tuberculosis-associated death of PLHIV**  Based on the random effect model of meta-regression using 22 studies. The overall pooled TB-associated mortality PLHIV was found to be 16 cases per 100 person-years (95% CI: 13.0- 19.2) with a significant heterogeneity (I^2^= 92.99%, P= 0.001) **(Figure 2).** | 10 |
| Synthesis of results | 21 |  | 10 |
| Risk of bias across studies | 22 | **Publication bias and Sensitivity analysis :** The publication bias was assessed using funnel plots, as well as quantitative methods such as Begg's and Egger's tests[47, 48]. The results indicated no evidence of publication bias and all studies were included in the plots, suggesting no significant publication biases The sensitivity analysis showed that all studies were within the confidence interval bounds of the meta-regression. Egger's (P=0.01) and Begg's tests (P=0.01) indicated no significant publication bias (Supplementary **Figure 2)** | 13 |
| Additional analysis | 23 |  | 19-21 |
| **DISCUSSION** | | |  |
| Summary of evidence | 24 | **Predictors for TB associated death of PLHIV**  In this systematic review and meta-analysis, adjusted odds ratios from primary studies were categorized into themes to identify the main risk factors for tuberculosis (TB) case fatality among people living with HIV (PLHIV). Eleven articles reported advanced WHO clinical stages (III&IV), four articles reported CD4 count, four articles reported missed isoniazid preventive therapy (IPT), and seven articles reported missed cotrimoxazole preventive therapy (CPT) as factors assessed for TB-associated death among PLHIV |  |
| Limitations | 25 | Among the highlighted strengths of this investigation were the utilization of a comprehensive search approach, precise inclusion criteria for the study population during outcome ascertainment, and the participation of three independent authors during the quality assessment of each study. Notwithstanding all of the previously mentioned advantages, this review's ability to determine TB case fatality rates is limited by a few studies with small sample sizes, the majority of which had design flaws that may have affected the results. These limitations should be taken into account before interpreting the findings |  |
| Conclusions | 26 | When compared to the World Health Organization's estimate of post-ART in Ethiopia in 2019, the pooled TB-associated mortality of PLHIV was overstated. Through random effect meta-regression, factors such as missed CPT and missed IPT, as well as progressed WHO clinical stages IV and III, were found. As a result, it is strongly advised to provide CPT and IPT concurrently and continuously with ART. Healthcare professionals should strictly inform patients on HIV and TB treatment options and should step up active TB screening during routine follow-ups |  |
| **FUNDING** There is no specific funding for this research | | |  |
