# Supplementary material for: Tuberculosis-associated mortality and risk factors for HIV-infected population in Ethiopia: a systematic review and meta-analysis
Source: Front Public Health. 2024 Jul 22;12:1386113. doi: 10.3389/fpubh.2024.1386113 (PMC11298472; doi:10.3389/fpubh.2024.1386113)
Supplement: SUPPLEMENTARY TABLE S3 — JBI critical appraisal checklists. [file Table_3.docx]

**Table S3: Quality appraisal result of included studies by** Joanna Briggs Institute (JBI) checklist

| **Author** | **Quality assessment questions** | | | | | | | | | | |  |  |  |
| --- | --- | --- | --- | --- | --- | --- | --- | --- | --- | --- | --- | --- | --- | --- |
|  | Two group employments at beginning | Exposure status ascertainment | Random selection of employment | Non response biases management | Data collection | Cases definition given | Strategies for in-complete follows- up management | Follow up time adequate | Confounding controlling | Outcome ascertainment at end of follow up | Was statically analysis correctly | Quality score | Quality status | Overall appraisal |
| Tadesse Sime et.al (1) | Y | Y | Y | UC | Y | Y | Y | Y | Y | Y | N | **3** | Low risk | Included |
| Debede Shewano et.al (2) | Y | Y | Y | Y | UN | Y | Y | Y | Y | Y | Y | **3** | Low risk | Included |
| [Assefa Tola](https://bmcpublichealth.biomedcentral.com/articles/10.1186/s12889-019-7980-x#auth-Assefa-Tola-Aff1) et.al (3) | Y | Y | Y | Y | UN | Y | Y | UN | Y | Y | Y | **2** | Low risk | Moderate |
| Asseffa Belayneh et.al(4) | Y | Y | Y | Y | Y | Y | Y | Y | Y | Y | Y | **3** | Low risk | Included |
| [Jifare Gemechu](https://pubmed.ncbi.nlm.nih.gov/?term=Gemechu+J&cauthor_id=34983618)(5) | Y | Y | Y | Y | Y | Y | Y | Y | Y | Y | Y | **3** | Low risk | Included |
| **Haile Abrha et.al (6)** | Y | Y | Y | Y | Y | Y | Y | Y | Y | Y | N | **3** | Low risk | Included |
| **M. Esayas et.al (7)** | Y | Y | Y | Y | Y | Y | Y | Y | Y | Y | Y | **3** | Low risk | Included |
| **Refera et.al (8)** | Y | Y | Y | UN | UN | Y | Y | Y | Y | UN | Y | **2** | Low risk | Moderate |
| **Birhan et.al (9)** | Y | Y | Y | Y | UN | Y | Y | Y | Y | Y | Y | **3** | Low risk | Included |
| **Eshete D. et.al (10)** | Y | Y | Y | Y | UN | Y | Y | Y | Y | Y | Y | **3** | Low risk | Included |
| **Weldegebreal et.al(11)** | Y | Y | Y | Y | UN | Y | Y | Y | Y | Y | Y | **3** | Low risk | Included |
| **Sileshi et.al (12)** | Y | Y | Y | Y | UN | Y | Y | Y | Y | Y | Y | **3** | Low risk | Included |
| **Emaby Gezae (13)** | Y | Y | Y | Y | UN | Y | Y | Y | Y | UN | Y | **3** | Low risk | Included |
| **Ahmed et.al (14)** | UN | Y | Y | Y | UN | Y | Y | Y | Y | UN | Y | **2** | Low risk | Moderate |
| **A.Teshome et.al (15)** | Y | Y | Y | Y | UN | Y | Y | Y | Y | Y | Y | **3** | Low risk | Included |
| **Dawit Z et. al(16)** | Y | Y | Y | Y | UN | Y | Y | Y | Y | Y | Y | **3** | Low risk | Included |
| **Alula et.al (17)** | Y | Y | Y | Y | UN | Y | Y | Y | Y | Y | Y | **3** | Low risk | Included |
| **Chanie ES, et.al(18)** | UN | Y | Y | Y | UN | Y | Y | Y | Y | UN | Y | **2** | Low risk | Moderate |
| **Wondimu et.al (19)** | Y | Y | Y | Y | UN | Y | Y | Y | Y | Y | Y | **3** | Low risk | Included |
| **Nigussie J. et.al(20)** | Y | Y | Y | Y | UN | Y | Y | Y | Y | Y | Y | **3** | Low risk | Included |
| **Seyoum et.al (21)** | Y | Y | Y | Y | UN | Y | Y | Y | Y | Y | Y | **3** | Low risk | Included |
| **Attale k. et.al (22)** | Y | Y | Y | Y | UN | Y | Y | Y | Y | Y | Y | **3** | Low risk | Included |

Key: Y=yes, N=no, UC=unclear, Q=Question

Grading of risk ofbiases for included articles was scored as follow

When score of articles is from 8 up to 11= will had (>75%) score, good quality , low risk of bias and included

When score of articles from 5 up to 7 = will had (50 to 75%) ,moderate quality ,low risk &artciles will be included

When score of artciles is from 3 upt o 5 = will had 25%to 50 %), high risk of biases and artciles will be not included

**References**

1. Sime T, Oljira L, Diriba A, Firdisa G, Gezimu W. Effect of active tuberculosis on the survival of HIV-infected adult patients who initiated antiretroviral therapy at public hospitals of Eastern Ethiopia: A retrospective cohort study. PLoS One. 2022;17(10):e0277021.

2. Worku2 DSaA. Tuberculosis treatment survival of HIV positive TB patients on directly observed treatment short-course in Southern Ethiopia: A retrospective cohort study. BMC Research Notes 2012, 5:682. 2012;<http://www.biomedcentral.com/1756-0500/5/682>.

3. Tola A, Mishore KM, Ayele Y, Mekuria AN, Legese N. Treatment Outcome of Tuberculosis and Associated Factors among TB-HIV Co-Infected Patients at Public Hospitals of Harar Town, Eastern Ethiopia. A five-year retrospective study. BMC Public Health. 2019;19(1):1658.

4. Belayneh M, Giday K, Lemma H. Treatment outcome of human immunodeficiency virus and tuberculosis co-infected patients in public hospitals of eastern and southern zone of Tigray region, Ethiopia. The Brazilian journal of infectious diseases : an official publication of the Brazilian Society of Infectious Diseases. 2015;19(1):47-51.

5. Gemechu J, Gebremichael B, Tesfaye T, Seyum A, Erkalo D. Predictors of mortality among TB-HIV co-infected children attending anti-retroviral therapy clinics of selected public hospitals in southern, Ethiopia: retrospective cohort study. Archives of public health = Archives belges de sante publique. 2022;80(1):11.

6. Hailay Abrha BT. Survival Experience and its Predictors among TB/HIV Co-infected Patients in Southwest Ethiopia. Epidemiology: Open Access. 2015;05(02).

7. Lelisho ME, Wotale TW, Tareke SA, Alemu BD, Hassen SS, Yemane DM, et al. Survival rate and predictors of mortality among TB/HIV co-infected adult patients: retrospective cohort study. Scientific reports. 2022;12(1):18360.

8. Wencheko2 HRE. Survival of HIV-TB co-infected adult patients under ART in Ambo Referral Hospital, Ethiopia. Ethiop J Health Dev 2013;27(2):88-93. 2013.

9. Birhan H, Derebe K, Muche S, Melese B. Statistical Analysis on Determinant Factors Associated with Time to Death of HIV/TB Co-Infected Patients Under HAART at Debre Tabor Referral Hospital: An Application of Accelerated Failure Time-Shared Frailty Models. HIV/AIDS (Auckland, NZ). 2021;13:775-87.

10. Gebreyes D. A survival analysis of prognostic determinant factors of time-to-death of HIV/TB co-infected patients under HAART followed-up in a public hospital in Ethiopia. HIV &amp; AIDS Review International Journal of HIV-Related Problems. 2023;22(2):110-30.

11. Weldegebreal F, Mitiku H, Teklemariam Z. Treatment outcome of tuberculosis among Human Immunodeficiency Virus positive patients in Eastern Ethiopia: a retrospective study. The Pan African medical journal. 2018;30:32.

12. Balewgizie Sileshi1* ND, Belaineh Girma3, Muluken Melese3 and Pedro Suarez4. Predictors of mortality among TB-HIV Co-infected patients being treated for tuberculosis in Northwest Ethiopia: a retrospective cohort study. BMC Infectious Diseases 2013, 13:297. 2013(<http://www.biomedcentral.com/1471-2334/13/297>).

13. Gebremeske KEGGHTAAK. Predictors of Time to Death among TB/HIV Co-Infected Adults on ART at Two Governmental Hospitals in Mekelle, Ethiopia, 2009-2016: A Retrospective Cohort Study. Annals of Infectious Disease and Epidemiology. 2020;2020 | Volume 5 | Issue 1 | Article 1049.

14. Ali SA, Mavundla TR, Fantu R, Awoke T. Outcomes of TB treatment in HIV co-infected TB patients in Ethiopia: a cross-sectional analytic study. BMC infectious diseases. 2016;16(1):640.

15. Teshome Kefale A, Anagaw YK. Outcome of tuberculosis treatment and its predictors among HIV infected patients in southwest Ethiopia. International journal of general medicine. 2017;10:161-9.

16. Dawit Z, Abebe S, Dessu S, Mesele M, Sahile S, Ajema D. Incidence and predictors of mortality among children co-infected with tuberculosis and human immunodeficiency virus at public hospitals in Southern Ethiopia. PLOS ONE. 2021;16(6):e0253449.

17. Teklu AM, Nega A, Mamuye AT, Sitotaw Y, Kassa D, Mesfin G, et al. Factors Associated with Mortality of TB/HIV Co-infected Patients in Ethiopia. Ethiopian journal of health sciences. 2017;27(Suppl 1):29-38.

18. Chanie ES, Gelaye GA, Tadesse TY, Feleke DG, Admas WT, Molla Alemu E, et al. Estimation of lifetime survival and predictors of mortality among TB with HIV co-infected children after test and treat strategies launched in Northwest, Ethiopia, 2021; a multicentre historical follow-up study. PLoS One. 2021;16(12):e0258964.

19. Wondimu W, Dube L, Kabeta T. Factors Affecting Survival Rates Among Adult TB/HIV Co-Infected Patients in Mizan Tepi University Teaching Hospital, South West Ethiopia. HIV/AIDS (Auckland, NZ). 2020;12:157-64.

20. Jemberu Nigussie MKGHHH. Predictors of Mortality Among Children Co-Infected with Tuberculosis and Human Immunodeficiency Virus in Region, North Ethiopia, Retrospective Follow-

Up Study. Biomed J Sci & Tech Res | BJSTR MSID006252. 2021.

21. Seyoum E, Demissie M, Worku A, Mulu A, Berhane Y, Abdissa A. Increased Mortality in HIV Infected Individuals with Tuberculosis: A Retrospective Cohort Study, Addis Ababa, Ethiopia. HIV/AIDS (Auckland, NZ). 2022;14:143-54.

22. Atalell KA, Birhan Tebeje N, Ekubagewargies DT. Survival and predictors of mortality among children co-infected with tuberculosis and human immunodeficiency virus at University of Gondar Comprehensive Specialized Hospital, Northwest Ethiopia. A retrospective follow-up study. PLOS ONE. 2018;13(5):e0197145.
